# Improvement of Metastatic Spinal Cord Compression After Decompression Surgery and Radiotherapy in a Patient Initially Treated for Rectal Cancer

**DOI:** 10.7759/cureus.21134

**Published:** 2022-01-11

**Authors:** Nobuko Utsumi, Hiromasa Kurosaki, Kosei Miura, Satoshi Baba, Yoshin Koyama

**Affiliations:** 1 Department of Radiation Therapy, Japan Community Healthcare Organization (JCHO) Tokyo Shinjuku Medical Center, Tokyo, JPN; 2 Department of Radiology and Radiation Oncology, Edogawa Hospital, Tokyo, JPN; 3 Department of Spinal Surgery, Japan Community Healthcare Organization (JCHO) Tokyo Shinjuku Medical Center, Tokyo, JPN; 4 Department of Surgery, Japan Community Healthcare Organization (JCHO) Tokyo Shinjuku Medical Center, Tokyo, JPN

**Keywords:** clinical case report, oligo-metastasis, rectum carcinoma, metastatic spinal cord compression, palliative radiation therapy

## Abstract

Many reports indicate that the prognosis of patients with rectal cancer who have thoracic spine metastases with spinal cord compression is poor. Here, we discuss a case of a patient who achieved an improvement of functional prognosis and long-term survival after undergoing surgery and radiotherapy.

We report a case of a 64-year-old female who was found to have metastatic spinal cord compression (MSCC) in the second thoracic vertebra, 10 years after surgery for rectal cancer. She experienced numbness in both legs and had gait difficulties. She underwent posterior decompression surgery and radiotherapy. Her neurological symptoms improved after radiotherapy, and the patient could maintain a standing position without assistance within one week after irradiation. She has since received adjuvant chemotherapy and continues to survive five years six months since MSCC onset.

## Introduction

Metastatic spinal cord compression (MSCC) is compression of the spinal cord, involving neuropathy, caused by the spread of a tumor to the vertebra. In addition to pain due to bone metastasis, patients with MSCC exhibit symptoms such as movement disorder due to muscle weakness, thermal hypoalgesia, sensory disorder, and bladder-rectal disorder. MSCC is one of the oncologic emergencies requiring prompt diagnosis and treatment to protect and improve nerve functions [1].

Aside from breast and prostate cancer, the vital prognosis for patients with MSCC is poor. In particular, several reports indicate that the prognosis for patients with MSCC due to rectal cancer is poor. Brown PD et al. reported that the median overall survival of 34 patients who received radiotherapy for spinal compression due to colorectal cancer metastasis was 4.1 months [2]. Leach MR et al. performed surgery on four patients with spinal metastasis due to colorectal cancer and reported that the mean survival was 15.3 months. This average included a patient still living at 57.1 months. The mean survival was just 1.3 months for the three patients who had expired [3].

## Case presentation

**Figure 1:**
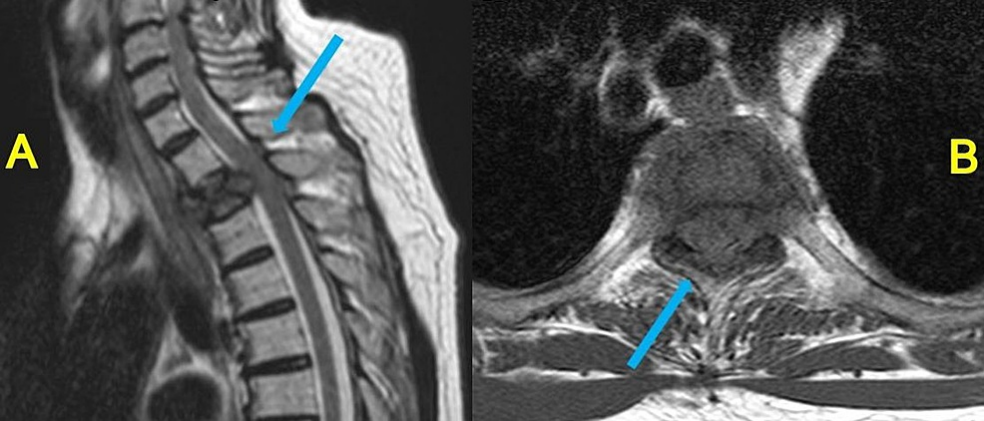
MRI at disease onset shows metastatic spinal cord compression in the second thoracic vertebra. A:　T2-weighted image (sagittal) B:　T1-weighted image (axial)

The patient was a 64-year-old woman diagnosed with rectal cancer (moderately differentiated adenocarcinoma, pT2N0M0: UICC-TNM classification version 6) who underwent low anterior resection (LAR). Liver metastasis was identified four years after surgery, and following chemotherapy using FOLFOX, excision for liver metastasis was performed. Liver metastasis was again identified eight years after LAR surgery, and the metastatic lesion was removed again. When lymph node metastasis and bone metastasis were identified 10 years after initial LAR, chemotherapy was considered, but we opted to observe the clinical progress on the patient's request. However, the patient developed numbness in both legs and gait difficulties, which were noted following the incidence of a fall. MRI showed spinal compression in the second thoracic vertebra (T2) (Figure 1). Neurologically, the manual muscle testing (MMT) score in both legs was 1, with thermal nociception diminishing below the T6-7 level.

**Figure 2:**
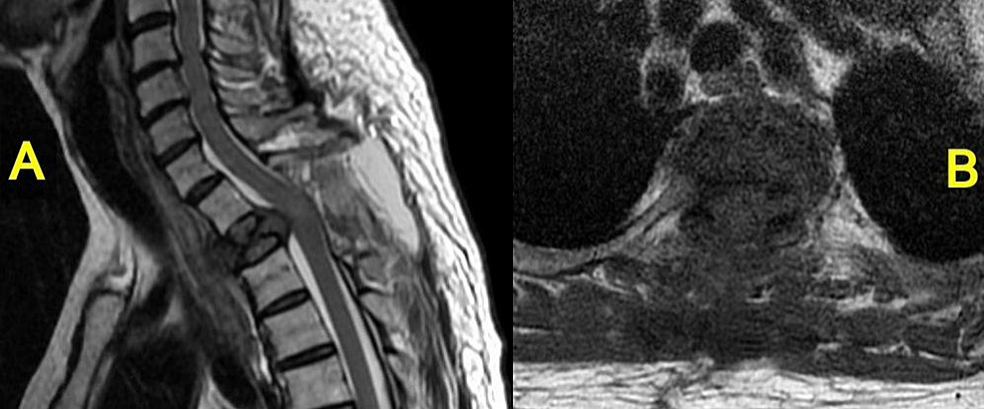
MRI on the day after start of radiotherapy (postoperative day 13). A:　T2-weighted image (sagittal) B:　T1-weighted image (axial)

Posterior decompression was carried out to address the MSCC at the T2 level. There was a temporary improvement of muscle strength in both legs, but the MMT score was reduced to 2, and thermal nociception diminished on postoperative day (POD) 10. She received radiotherapy at 30 Gy/10 fractionation on POD 12. MRI taken on the day after beginning radiotherapy is shown in Figure 2. The patient underwent rehabilitation treatment and radiotherapy simultaneously, eventually showing improvement of neurological symptoms by the end of treatment. This allowed her mobility with the use of a wheelchair.

**Figure 3:**
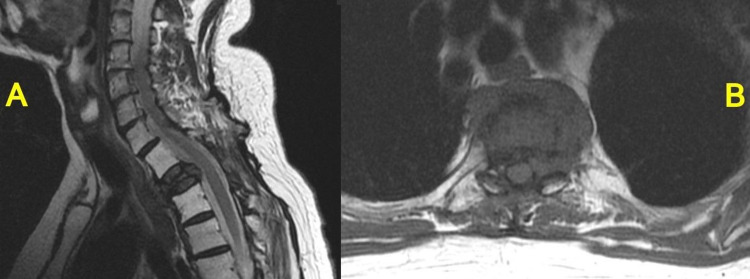
MRI five years two months after irradiation shows no recurrence. A:　T2-weighted image (sagittal) B:　T1-weighted image (axial)

The patient continued her rehabilitation treatments and became capable of maintaining a standing posture without assistance one week after radiotherapy. One month after completion of radiotherapy, the patient was able to walk several meters using a walking aid and was eventually capable of unassisted gait one year after treatment. Her general condition was good, leading her to receive chemotherapy for around three years (34 courses of IRIS + Avastin and four courses of CPT-11 monotherapy). Para-aortic lymph node metastasis appeared five years after radiotherapy of the thoracic spine. To treat this, the patient received 56 Gy/28 fractionation of radiotherapy combined with surgery and chemotherapy. Figure 3 shows the patient's MRI five years two months after radiotherapy for MSCC. There has been no recurrence of metastatic lesions in the thoracic spine after radiotherapy, and the patient continues to survive five years six months after MSCC onset.

## Discussion

Malignancy-induced spinal compression occurs in 5% of all cancer patients and is most common in breast cancer. It is regarded as an oncologic emergency because a delayed start of treatment can lead to neurological symptoms such as paralysis. The condition is treated by surgery, steroidal drugs, radiotherapy, or a combination of these modalities. However, the vital prognosis is still poor in many cases. Patchell RA et al. conducted a randomized controlled trial involving 101 patients with spinal compression, comparing a radiotherapy arm against spinal decompression + adjuvant radiotherapy arm, and reported the significant improvement of functional prognosis and vital prognosis [4]. This is because it takes several days for radiotherapy to be effective for secondary vascular injury associated with spinal cord infarction due to compression on the spinal cord. At the same time, surgical decompression has the benefit of imparting an immediate therapeutic effect. The same study states the conditions for eligibility for the treatment to be:

(1) Within 48 hours of paraplegia onset;

(2) Only one culprit lesion (regardless of presence or absence of spinal metastasis not causing spinal compression);

(3) Patients having a general condition that allows them to tolerate surgery;

(4) Patients having a vital prognosis of three months or longer.

Since spinal decompression alone does not have an antitumor effect, it is not a treatment capable of addressing long-term tumor control; therefore, adjuvant radiotherapy is still necessary [5].

This patient had oligometastases at the time of MSCC onset. Oligometastases or oligo-recurrence is a condition in which patients have a limited number of distant metastases or recurrences. Hellman S and Weichselbaum RR published an editorial regarding oligometastases in 1995 [6]. It did not discuss primary lesions but suggested that if there are only a few distant metastases, adding local treatment to the sites of distant metastases may prolong the prognosis. Later, Niibe Y et al. suggested that radiation therapy for isolated para-aortic lymph node recurrence in cervical cancer could significantly impact survival [7,8]. In 2012, synchronous oligometastases were defined as having 1-5 metastatic or recurrent lesions, and oligo-recurrence was defined as having 1-5 metastatic lesions without recurrence of the primary lesion [9].

Recurrence of oligometastases or oligo-recurrence and local control of metastatic lesions may improve patient survival [10,11]. Advances in chemotherapy and targeted molecular drugs and the development of immune checkpoint inhibitors have improved the prognosis of cancer patients with recurrence and metastasis. Therefore, when determining the appropriate treatment strategy, it is essential to note the presence of one to five metastatic lesions.

For this patient, 30 Gy/10 fractionation was prescribed as palliative radiation at the time of MSCC onset. Regarding dose fractionation in radiotherapy, while single irradiation is said to maintain motor function and bladder function at the same level as multiple irradiations, some reports indicate that higher doses provide a higher local control rate. In recent years, stereotactic radiotherapy (SRT) is also being used [5,12]. Moulding HD et al. studied 21 patients who underwent SRT after spinal decompression and fixation and reported that 24 Gy/1 fraction controlled the tumor regardless of the tumor's radiation sensitivity [5]. Laufer I et al. studied 186 patients who underwent SRT after surgery and reported that high-dose prescription could control radiation-resistant tumors that cannot be controlled well with normal dose fractionation [13]. In the future, we believe SRT may also be used for cases of oligometastases or oligo-recurrence [14]. Our patient had a case of rectal cancer with a tumor that was not highly sensitive to radiation. This may be considered an indication for SRT. However, when the patient underwent radiotherapy in 2015, insurance coverage had not been approved for SRT in Japan. As of April 2020, insurance coverage now applies for SRT performed for oligometastases in Japan.

For this patient, surgery was performed the day after her fall. While her neurological symptoms initially improved temporarily, the symptoms deteriorated on POD 10. According to the report by Patchell RA et al., radiotherapy was supposed to be performed within 14 days of surgery, and within one month of surgery, according to the report by Tancioni F et al. However, our patient received radiotherapy starting POD 12 owing to the deterioration of her symptoms [4,15]. Our observations suggest that radiotherapy should be considered without delay when symptoms deteriorate.

## Conclusions

Aside from breast and prostate cancer, the prognosis of many metastatic spinal cord compression cases tends to be poor. But owing to the advancement of systemic treatment, including chemotherapy, targeting therapy, and immunotherapy, we now see long-term survival in these patients. Our patient started radiotherapy on day 12 after posterior decompression and showed improvement of neurological symptoms. Our case report suggested that long-term survival and good quality of life are achievable even in some MSCC patients with stage 4 rectal cancer.

## Appendices

Patient's perspective: Thanks to radiotherapy, I am able to walk now, which feels like a dream. Thanks to this, I am able to enjoy traveling abroad and going hiking. I should take advantage of this and live another 20 years!
